# Supplementary material for: Bta-miR-206 and a Novel lncRNA-lncA2B1 Promote Myogenesis of Skeletal Muscle Satellite Cells via Common Binding Protein HNRNPA2B1
Source: Cells. 2023 Mar 27;12(7):1028. doi: 10.3390/cells12071028 (PMC10093610; doi:10.3390/cells12071028)
Supplement: Supplementary file 1 [file cells-12-01028-s001.zip › cells-2132393-supplementary/cells-2132393 Supplementary for XML.pdf]

Supplementary

Table S1. The protein-coding potential.

#ID	peptide_length	Fickett_score	pI	ORF_integrity	coding_probability	label
LncA2B1	46	0.3262	9.0211	1	0.0158	noncoding
Linc-MD1	99	0.3399	10.1044	−1	0.1648	noncoding
HPRT1	219	0.4637	6.2108	1	0.9997	coding

Table S2. siRNAs sequences for RNA knockdown.

Fragment name	Sequences/（5′→3′）
si-lncA2B1	CAACATTCCATGACATGAA
si-HNRNPA2B1	GAGGGATCCTGCAAGCAAA
si-DHX9	GGACCTCAAGAATGAGTTA

Table S3. Primers for PCR.

Genes	Primer sequences/（5′→3′）	Product length
TCONS_00048554 (lncA2B1-1)	F: TGTGGCTACCTAATTTGCTTGT<br>R: ACTTGCAGAAAACTGCCATAGG	92 bp
TCONS_00048554 (lncA2B1-2)	F: TGTGACTTGTTTGGCCGGTA<br>R: TCTTCATACTGGCACCTCCTC	84 bp
TCONS_00070803	F: GAGGCAGGACAACCCATCTT<br>R: GGGACAGTATGAGGTAGGGGAG	136 bp
TCONS_00024351	F: GAGCGAGTACATGGGAGGAA<br>R: TTAGACTCCCCCTCCACACA	112 bp
TCONS_00085765	F: TTCAGTGTAGTCGCTGGCAC<br>R: GCCCCTTCCATCAGCACATT	128 bp
TCONS_00109062	F: CTTCTCTCCTACTTCACAGCCC<br>R: GAGAAGGCAAATGGGGTCAG	90 bp
TCONS_00042276	F: CAAGGGGTCACGATGTCTCA<br>R: GGAGAAACCACCGACCAACC	107 bp
Pax3	F: CAAAAGAGAGAACCCCGGCA<br>R: ACTCACTGAGGGTACGGTGT	94 bp
Pax7	F: AGCCAGAGTTTCAACGGGAG<br>R: GTCGCCAACAGACAACACAC	93 bp
HDAC4	F: AAGCCAAATGCTTTCTCTGTCC<br>R: GTGCTGGGCATGTGGTTCAC	97 bp
MyoG	F: GGCTGACAAATGCCAGACTATCC<br>R: TGGTCCCTTGCTTTATCTCCCT	140 bp
MyHC	F: CTGGAATCCGGAGGCAGAA<br>R: TTTTCGAAGGTAGGGAGCGG	105 bp
GAPDH	F: TGTTGTGGATCTGACCTGCC<br>R: AAGTCGCAGGAGACAACCTG	135 bp
pre-GAPDH	F: TCCCTCCACGATGCCAAAG<br>R: GAAGCGGGCTGATAGTGCC	143 bp
β-actin	F: CATGTACGTTGCTATCCAGGC<br>R: CTCCTTAATGTCACGCACGAT	250 bp
U6	F: GGAACGATACAGAGAAGATTAGC<br>R: TGGAACGCTTCACGAATTTGCG	68 bp
HNRNPA2B1	F: ATGGAGGAGGAAGAGGAGGATATGG<br>R: AGTTGTCATAACCACCTCCGTAGC	84 bp
DHX9	F: TGGAGGCTCTGGTTGTTGAAGT	90 bp

	R: GGCGGATTGTGTTCAGCATACG	
bta-miR-206 [a]	F: CGTGGAATGTAAGGAAGTGTGTGG	
5.8s-rRNA [a]	F: GACTATGCCTGTCTGAGCGTCACTT	
LncA2B1 (52–269bp)	F1: TTTGGCCGGTAGGAACGTAG<br>R1: TGTGGTGTTTCACGCCATGT	237 bp
LncA2B1(141–394bp)	F2: TAAGGATTCCAGGGCAGTGT<br>R2: CAGAGCCTTTTCAACCAGGAA	254 bp
LncA2B1 (375–878bp)	F3: TCCTGGTTGAAAAGGCTCTGG<br>R3: GGAAGCGATGGTATGCTGGA	504 bp
LncA2B1 (202–2005bp)	F4: CAGTGGCTTGGCAACTTCAC<br>R4: GCAGAAAACTGCCATAGGCATC	1,804 bp
LncA2B1 (104–2005bp)	F5: GAAGAGGAGGTGCCAGTATGA<br>R5: GCAACTTGCAGAAAACTGCCATAGG	1,902 bp
LncA2B1 Sense	F:TAATACGACTCACTATAGGGGGCAAGAGAAG<br>TAACACATGACATCTTTCT<br>R:CAAAATTGAAAATGATCATGTTATAAGAAACT	2,033 bp
LncA2B1 anti-Sense	F:TAATACGACTCACTATAGGGCAAAATTGAAAA<br>TGATCATGTTATAAGAAACT<br>R: GGCAAGAGAAGTAACACATGACATCTTTCT	2,033 bp

[a] Sense primers for mature miRNAs and 5.8 s rRNA RT-qPCR are provided here; GeneCopoeia were provided anti-sense primers as a Universal Adaptor PCR Primer.

Table S4. The primer sequences of gene cloning.

Genes	Primer sequences/（5′→3′）	Sites
pET-lncA2B1	F: CCGGAATTCGAAGAGGAGGTGCCAGTATGAA	EcoRI
	R: CCGCTCGAGCGCTGATTCCAGAGCCTTTTC	XhoI
pcDNA3.1-lncA2B1	F: CCGCTCGAGGGCAAGAGAAGTAACACATGACATCTTTCT	XhoI
	R: TGCTCTAGACAAAATTGAAAATGATCATGTTATAAGAAACT	XbaI
psicheck2-lncA2B1	F: CCGCTCGA**G**GGCAAGAGAAGTAACACATGACATCTTTCT	XhoI
	R: ATTTGCGGCCG**C**CAAAATTGAAAATGATCATGTTATAA-GAAACT	NotI

Table S5. The antibody information.

Name	Dilution	Manufacturer
Myosin heavy chain (MyHC)	1:100	DSHB, USA
Myogenin	1:500	ZSGB-BIO, China
GAPDH	1:1,000	
HRP Goat anti-mouse IgG	1:20,000	
HRP Goat anti-rabbit IgG	1:20,000	
Anti-PAX7 Antibody	1:1,000	Bioss, China
Anti-HDAC4 antibody	1:1,000	
Anti-Myosin VIIa	1:500	abcam, USA
Anti-HNRNPA2B1 rabbit polyclonal anti-body	1:500	BBI, China
Anti-DHX9 Antibody	1:100	BOSTER, China
