# Supplementary material for: Bta-miR-206 and a Novel lncRNA-lncA2B1 Promote Myogenesis of Skeletal Muscle Satellite Cells via Common Binding Protein HNRNPA2B1
Source: Cells. 2023 Mar 27;12(7):1028. doi: 10.3390/cells12071028 (PMC10093610; doi:10.3390/cells12071028)
Supplement: Supplementary file 1 [file cells-12-01028-s001.zip › cells-2132393-supplementary/S1 sequence.pdf]

Supplementary

The sequence of Bta-miR-206

UGGAAUGUAAGGAAGUGUGUGG

The sequence of lncA2B1

GGCAAGAGAAGTAACACATGACATCTTTCTCTCAAATTAAAATGTGACTT-
GTTTGGCCGGTAGGAACGTAGTGGGAGATGGAAAAGACCGGGAG-
TCGAAAAGGAAGAGGAGGTGCCAGTATGAA-
GATGACTCAACAGAAGTAAGGATTCCAGGGCAGTGTCAGATCTGAAAAA-
GCAATTCCAGTACCAGTTCCTTGTCTCACAGTGGCTTGGCAACTTCAC-
CTCTCTCAAAGTAGAGAG-
GAAAATCAAGTCAAACAGGCCTTCCTGAAACATGGCGTGAAACAC-
CACATGTCACCTTTGTTCCTGGAGGAGACACTAAACAAGTACTTGTTGAGAA-
GAAGAAATGATCCTGAAGAGAAAGCTGCATAAAGTATTTCCTGGTT-
GAAAAGGCTCTGGAATCAGCGTCTAGAATTTGAATCTCAACTCTGAAGTG-
CATCAGCTGTGTGACCTTAGGAACTCTCGGCTGGATTTGTGACTTGCTTGAA-
GAAACAGAATGTGATGGATGTGAAGCTGTGTGACTTTCAGCTTTTT-
GAAACATAATTAAAAGTCATGATTTAGAGCATTTGCCAATTTCTAA-
GAATCCACCTGAAATGCAGAAGACACAGGAAATGTAGGTTT-
GATCCCTAGGTCAGGAAGACCCCCTGTAGAGGAAATGGCAACACACTCCAG-
TGTGCTTGCCTGAAAAATCCTGTGGACAAAGGAGCCTAGTGGGC-
TATGGTCCAAAGGGTTGCAAAGAGTTGGACATGACTGAATAACTGAG-
CACATGGTGTAAGTGCTGCCAGCATGACTGATTTCGAGCTACCAATGTGA-
TATCACAGAGCATGTAGTTGGAAAGAGATAGCATATATGAC-
CAATGGGCTCCAGCATACCATCGCTTCCAGACTTACTCCTTAAGA-
GATCTGTCAGCTTCCATTTTTGCTTTCTTGGAATCCAGTT-
GACATGAAGGATCCTTGGGCTAGGCTTTTGAATGATGA-
GAGGCCAAATGGAAAGAAAGGCCATGTGGAAAAGAAAAGAA-
GCCTTAGGCGATCCATCCCTAGCTGGGCTCCCAGCTGAGTGCAGCTG-
CATGCTTGGCCTCAGCCAACATTCCATGACATGAAAGACCAC-
GCAGCCAACTCACAGAAATGTGAGACACAATACATTGTTGTAGATTTAA-
GCGACTACATTTTGGAATATTTTAATTAGTCAGCAATAGGTAACTG-
GAAACATGTATTGCCAAGCCATTGTAAAACAATTTTCACATTTTACTCC-
TATGTTAGCAACGTGGCTCAGCTGGTAAAGAATCTTATCTGCAATGTGGGA-
GACCTGGGAAGATCCCCTGAAGAAGGGAAAGGCTACCCACTCCAGTATTTT-
GGCCTGGAGAATTCCATGGACTTTATAGTCCGTGGGGTCTCACAGAGTCAGA-
CATGTCTAAGCAACTTTCACTTTCACTAGCAATGTACAAGAGTGCCTT-
GTTCTTTGCACATCAGCCTGCACTTTGCATTATCATTCACCAGGTTGA-
TAAGTTAAAAAATGTACTTTGCAGTTTAGCTTGTATTCTTTGAAAATGAATTT-
GAATAGTTTTTCAGATGCTTATTGGCCATAAATT-
GGCTGTGCTCTCTGTTCATTTTTCTCTTGATTTGTAAGGGCTCTTTATATTTTAA-
TAGGGCTTCTCAGGTGGCTCAATGGTAAGTTATCTGCCTGCAGTGTGGAAGA-
CACAGGTTTGATACCTGGGTCAGGAATATCGCCTGGAGAAGGAAATGG-
CAACCCGCTGTAGTATTGTTGCCTGGGAAATACCAAGGACAGAGGAGCCTGG-
CAGGCTGTAGCCCATGGGATCACAAAATAATCAGACATGACTTAGCGAG-
TAAACAAAAACAATATAGTTGGTTAACAATTTTGTG-
TATAGTAACAACAAATGGACAATATTTTGCAATGTATGTGGCTACCTAATTT-
GCTTGTATGTTCTTTGCAGTAAAAAGCTTAAACTATTT-
GTATGAAAAAATGATGCCTATGGCAGTTTTCTG-
CAAGTTTCTTATAACATGATCATTTTCAATTTTG
